# Association of Infectious Disease Physician Approval of Peripherally Inserted Central Catheter With Appropriateness and Complications

**DOI:** 10.1001/jamanetworkopen.2020.17659

**Published:** 2020-10-21

**Authors:** Valerie M. Vaughn, Megan O’Malley, Scott A. Flanders, Tejal N. Gandhi, Lindsay A. Petty, Anurag N. Malani, Allison Weinmann, Jennifer K. Horowitz, Vineet Chopra

**Affiliations:** 1 Division of Hospital Medicine, Department of Internal Medicine, University of Michigan Medical School, Ann Arbor; 2 Center for Clinical Management Research, Veterans Affairs Ann Arbor Healthcare System, Ann Arbor, Michigan; 3 Patient Safety Enhancement Program, Veterans Affairs Ann Arbor Healthcare System, Ann Arbor, Michigan; 4 Division of Infectious Diseases, Department of Internal Medicine, St Joseph Mercy Health System, Ann Arbor, Michigan; 5 Division of Infectious Diseases, Department of Internal Medicine, Henry Ford Hospital, Detroit, Michigan

## Abstract

**Importance:**

Peripherally inserted central catheters (PICCs) are frequently used to deliver intravenous antimicrobial therapy. However, inappropriate PICC use may lead to patient harm.

**Objective:**

To evaluate whether infectious disease physician approval prior to PICC placement for intravenous antimicrobials is associated with more appropriate device use and fewer complications.

**Design, Setting, and Participants:**

This cohort study of 21 653 PICCs placed for a primary indication of intravenous antimicrobial therapy between January 1, 2015, and July 26, 2019, was conducted in 42 hospitals participating in a quality collaborative across Michigan among hospitalized medical patients.

**Main Outcomes and Measures:**

Appropriateness of PICCs was defined according to the Michigan Appropriateness Guide for Intravenous Catheters as a composite measure of (1) single-lumen catheter use, (2) avoiding use of PICCs for 5 days or less, and (3) avoiding use of PICCs for patients with chronic kidney disease (defined as an estimated glomerular filtration rate <45 mL/min/1.73 m^2^). Complications related to PICCs included catheter occlusion, deep vein thrombosis, and central line–associated bloodstream infection. The association between infectious disease physician approval, device appropriateness, and catheter complications was assessed using multivariable models, adjusted for patient comorbidities and hospital clustering. Results were expressed as odds ratios with 95% CIs.

**Results:**

A total of 21 653 PICCs were placed for intravenous antimicrobials (11 960 PICCs were placed in men [55.2%]; median age, 64.5 years [interquartile range, 53.4-75.4 years]); 10 238 PICCs (47.3%) were approved by an infectious disease physician prior to placement. Compared with PICCs with no documented approval, PICCs with approval by an infectious disease physician were more likely to be appropriately used (72.7% [7446 of 10 238] appropriate with approval vs 45.4% [5180 of 11 415] appropriate without approval; odds ratio, 3.53; 95% CI, 3.29-3.79; *P* < .001). Furthermore, approval was associated with lower odds of a PICC-related complication (6.5% [665 of 10 238] with approval vs 11.3% [1292 of 11 415] without approval; odds ratio, 0.55; 95% CI, 0.50-0.61).

**Conclusions and Relevance:**

This cohort study suggests that, when PICCs were placed for intravenous antimicrobial therapy, infectious disease physician approval of PICC insertion was associated with more appropriate device use and fewer complications. Policies aimed at ensuring infectious disease physician approval prior to PICC placement for antimicrobials may improve patient safety.

## Key Points

**Question:**

Is approval by an infectious disease physician prior to placement of a peripherally inserted central catheter (PICC) for intravenous antimicrobials associated with appropriate device use or complications?

**Findings:**

In this cohort study of 21 653 PICCs placed for intravenous antimicrobials in 42 hospitals, 47% of PICCs were placed with approval of an infectious disease physician. Compared with nonapproved PICCs, approved PICCs were more likely to be appropriately placed, and the patients less likely to experience complications.

**Meaning:**

This cohort study suggests that infectious disease physician approval of PICCs prior to placement for intravenous antimicrobial therapy is associated with more appropriate device use and fewer complications.

## Teaser

This cohort study evaluates whether infectious disease physician approval prior to placement of a peripherally inserted central catheter (PICC) for intravenous antimicrobials is associated with more appropriate device use and fewer complications.

## Introduction

Peripherally inserted central catheters (PICCs) are central venous catheters used ubiquitously in medical care. Compared with central venous catheters placed in the neck or chest, PICCs are placed in upper-extremity veins in adults and consequently avoid many of the immediate insertion complications associated with device placement. As PICC use has increased, however, reports of complications, such as deep vein thrombosis (DVT) and central line–associated bloodstream infection (CLABSI), have emerged.^1^ One strategy to reduce the risk of PICC-related complications is to consider the appropriateness of PICC use, including aspects such as device characteristics and use of alternatives. For example, use of single-lumen (vs multilumen) devices can reduce the risk of DVT or CLABSI.^2,3^ Similarly, use of PICCs with a smaller cross-sectional diameter (ie, catheter gauge) is known to be associated with fewer thrombotic complications.^4^ Furthermore, because safer alternative devices are available,^5^ PICC use should be avoided if access is needed for a brief period (ie, ≤5 days) or for patients with chronic kidney disease.^6,7^ Collectively, these findings have led to Choosing Wisely recommendations^8^ and appropriateness criteria^9^ to inform and benchmark PICC use.

One of the most common indications for PICC insertion is intravenous antimicrobial therapy. Consequently, infectious disease (ID) physicians are often involved in decision-making related to PICC use. If they are consulted early, ID physicians may be able to prevent inappropriate PICC use—for example, for patients who could have their antimicrobials discontinued or switched to oral therapy. Similarly, because ID physicians often treat CLABSIs, they may be more likely to recommend interventions to reduce the risk of CLABSI, such as the use of single-lumen PICCs or antimicrobial catheters in patients who are at high risk of adverse events.^10^ Despite this, little is known about the association of ID physician approval with PICC appropriateness and outcomes. Because device complications are common in patients receiving intravenous antimicrobials,^11^ there may be an opportunity to improve patient safety by engaging ID physicians prior to PICC insertion.

The Michigan Hospital Medicine Safety Consortium (HMS) is a statewide quality collaborative aimed at preventing adverse events in hospitalized medical patients. Since 2015, the HMS has collected detailed data on PICCs, allowing for assessment of PICC appropriateness, and patient outcomes. We sought to evaluate whether ID physician approval of PICC use prior to placement for intravenous antimicrobials is associated with appropriate device use and fewer complications.

## Methods

### Study Setting and Participants

This prospective cohort study included patients who had a PICC inserted between January 1, 2015, and July 26, 2019, in 42 diverse Michigan hospitals participating in the HMS (a collaborative quality initiative funded by Blue Cross Blue Shield of Michigan). Hospital participation in the HMS is voluntary and includes almost half of all nongovernment hospitals in Michigan, including rural hospitals, community hospitals, and academic teaching hospitals.^12^ Sampling, inclusion, and exclusion criteria have been described previously.^6,13^ In brief, the HMS collects data on adult medical patients admitted to a general ward or an intensive care unit within a participating hospital who had a PICC placed for any reason during the course of clinical care. Patients are excluded if they are younger than 18 years, pregnant, admitted to a nonmedical service (ie, surgery), or admitted for observation. Data from patients are sampled at each facility in 2-week intervals, with 17 medical (intensive or general care) patients who received PICCs included in each wave at each hospital. This study followed the Strengthening the Reporting of Observational Studies in Epidemiology (STROBE) reporting guidelines. Because the purpose of the HMS is to measure and improve the quality of existing practice, this project received a “not-regulated” status from the University of Michigan Institutional Review Board; all patient data were deidentified.

For this analysis, we were specifically interested in patients for whom the primary indication for PICC insertion was documented as intravenous antimicrobial therapy. Thus, PICCs were included only if they had an indication for intravenous antimicrobials documented in the physician order, vascular access insertion note, or interventional radiology procedure note. Peripherally inserted central catheters for which the primary indication included other reasons (eg, difficult access or chemotherapy) were excluded.

### Data Collection

Trained data abstractors at each hospital collected detailed information from medical records using a standardized template including patient data, clinician data (eg, indication for insertion or consultants), and device characteristics (eg, number of lumens). Hospitals undergo random quality assurance checks to verify accuracy. In addition, data on PICC complications, including catheter occlusion, symptomatic DVT, and CLABSI, were recorded using standardized definitions^14^ and confirmed by medical record review. After PICC placement, all patients were followed up prospectively until PICC removal, 30 days, or death.

### Outcomes

The primary outcome of interest was the appropriateness of PICC use, assessed based on the Michigan Appropriateness Guide for Intravenous Catheters.^9^ Accordingly, each PICC placement for antimicrobial therapy was evaluated according to the following 3 appropriateness metrics: (1) use of single-lumen rather than multilumen catheters, (2) use of PICCs for more than 5 days (as opposed to ≤5 days), and (3) avoiding PICC use in patients with chronic kidney disease (CKD; estimated glomerular filtration rate <45 mL/min/1.73 m^2^). Therefore, PICCs were considered fully appropriate if the device was single-lumen, in place for more than 5 days, and inserted in a patient without CKD. Peripherally inserted central catheters that did not meet 1 or more of these criteria were considered inappropriate.

In addition to catheter appropriateness, we assessed major complications associated with PICC use, including CLABSI, venous thromboembolism, and catheter occlusion. A PICC-related bloodstream infection (ie, CLABSI) was defined in accordance with the Centers for Disease Control and Prevention and National Healthcare Safety Network criteria.^15,16^ Venous thromboembolism was defined as clinically diagnosed upper-extremity DVT and/or pulmonary embolism in a symptomatic patient that was confirmed via imaging (ultrasonography or venograpahy for DVT and computed tomography, ventilation perfusion scan, or pulmonary angiography for pulmonary embolism).^13^ Peripherally inserted central catheter occlusion was defined as a temporary or permanent inability to aspirate blood or infuse therapeutic agents, necessitating use of tissue plasminogen activator.^17^ To ensure reliability and integrity of definitions, all documented complications were manually audited.

### Variables

We were primarily interested in whether ID physician approval of PICCs was associated with more appropriate catheter use and fewer complications from PICC insertion for intravenous antimicrobials. We ascertained ID physician approval of PICC use by examining consultation or primary notes for specific verbiage supporting PICC insertion (eg, “OK to place PICC per ID”). To further explore variation in ID physician approval, we also gathered information from participating hospitals on whether ID physician consultation was available onsite or whether access to ID physicians occurred via remote consulting. Finally, we also evaluated general hospital characteristics (eg, bed size), sourcing data from an annual quality survey filled out by a physician or quality champion and data abstractor at each participating hospital.

### Statistical Analysis

We compared PICC appropriateness and complications by ID physician approval by fitting logistic mixed-effect models with hospital-specific random effects after adjusting for patient characteristics (age, sex, body mass index, race/ethnicity, and Charlson Comorbidity Index score), hospital characteristics (number of beds, profit status, and teaching status), and year of PICC placement. There were minimal missing data, and we had outcome data on all but 3 patients (4958 had 14-day but not 30-day follow-up information). Results were expressed as raw proportions and unadjusted or adjusted odds ratios (ORs) with 95% CIs. To ensure rigor and to take into account inflation that may occur when the incidence of events was low, unadjusted and adjusted relative risk ratios with 95% CIs were also estimated. All pairwise comparisons were made using *t* tests for continuous data and χ^2^ tests for categorical data. To explore hospital variability, we also report the percentage of PICCs that were approved by ID physicians with 95% CIs (after excluding hospitals with <25 PICCs). For the primary analysis, we conducted sensitivity analyses in which we (1) report risk ratios and (2) included only the first PICC placed per patient (removing 1126 PICCs placed in previously included patients). All *P* values were from 2-sided tests, and results were deemed statistically significant at *P* < .05.

## Results

**Figure 1.**
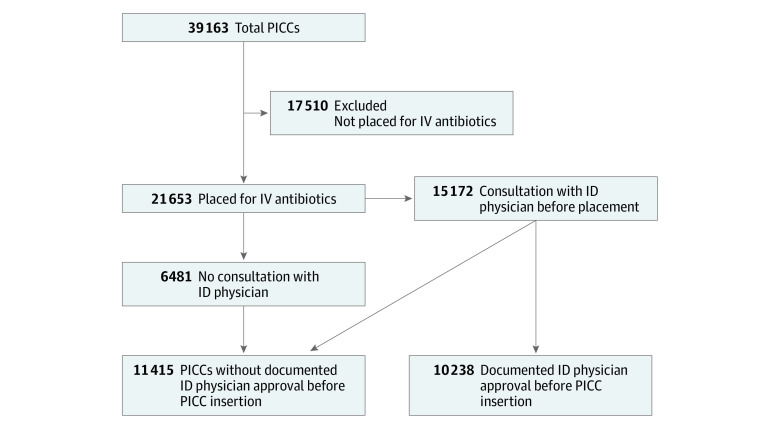
Patient Inclusion Flow Diagram ID indicates infectious disease; IV, intravenous; and PICC, peripherally inserted central catheter.

Of 39 163 PICCs placed across HMS hospitals between January 1, 2015, and July 26, 2019, 21 653 (55.3%) had a primary indication of intravenous antimicrobials and were included in this analysis (Figure 1). Included patients were predominantly White (16 451 PICCs [76.0%] placed in White patients) and male (11 960 PICCs [55.2%] placed in men), with a median age of 64.5 years (interquartile range, 53.4-75.4 years) (Table 1). Of the 21 653 included PICCs, 17 904 (82.7%) had antimicrobials as the only indication for placement. Of the 21 653 PICCs with an indication for intravenous antimicrobials, 15 172 (70.1%) were placed after a consultation with an ID physician; 6481 (29.9%) were not. Of the 15 172 PICCs placed after a consultation with an ID physician, documented approval by the ID physician for PICC insertion was noted for 10 238 patients (67.5%). Thus, of the 21 653 PICCs placed for intravenous antimicrobials, 10 238 (47.3%) had approval from an ID physician documented prior to device placement. The patient and PICC characteristics of patients with vs without ID physician approval are shown in Table 1. Peripherally inserted central catheters placed outside of the intensive care unit, that had antimicrobials as the only indication, or with a longer catheter dwell time were more likely to have documented ID physician approval. Consultations with ID physicians took place a mean of 1.5 days prior to PICC placement, and PICCs were placed a mean of 5.6 days (median, 3 days) prior to hospital discharge.

**Table 1.** Characteristics of PICCs With or Without ID Physician Approval

Characteristic	PICCs, No. (%)		*P* value
	With approval (n = 10 238)	Without approval (n = 11 415)	
**Patient characteristics**			
Race/ethnicity			
White	8127 (79.4)	8324 (72.9)	<.001
Black	1597 (15.6)	2478 (21.7)	<.001
Asian	61 (0.6)	63 (0.6)	.67
Other	184 (1.8)	222 (1.9)	.42
Unknown	163 (1.6)	189 (1.7)	.71
Sex			
Male	5856 (57.2)	6104 (53.5)	
Female	4382 (42.8)	5311 (46.5)	<.001
Age, median (IQR), y	64.5 (53.4-75.3)	64.6 (53.4-75.5)	.23
Charlson Comorbidity Index, median (IQR)	3 (1-5)	3 (1-5)	<.001
eGFR ≥45 mL/min/1.73 m^2^	1230 (12.0)	1755 (15.4)	<.001
In intensive care unit at the time of PICC insertion	345 (3.4)	2360 (20.7)	<.001
**PICC characteristics**			
Antimicrobial is only indication	9411 (91.9)	8493 (74.4)	<.001
Multiple indications for PICC placement	827 (8.1)	2922 (25.6)	<.001
Single-lumen device	8908 (87.0)	6820 (59.7)	<.001
Dwell time, median (IQR), d	20 (12-34)	13 (7-25)	<.001
**Hospital characteristics**			
Number of beds, median (IQR)	310 (217-458)	383 (255-573)	<.001
Hospital profit type			
For profit	561 (5.5)	800 (7.0)	<.001
Nonprofit	9007 (88.0)	9621 (84.3)	<.001
Academic hospital	5394 (52.7)	6868 (60.2)	<.001
ID physician consultation availability^a^			
On site	7691/8834 (87.1)	8719/10 501 (83.0)	<.001
Visiting or available for remote consultation	955/8834 (10.8)	1479/10 501 (14.1)	<.001
Unavailable	188/8834 (2.1)	303/10 501 (2.9)	<.001

Abbreviations: eGFR, estimated glomerular filtration rate; ID, infectious disease; IQR, interquartile range; PICC, peripherally inserted central catheter.

^a^ Data on ID physician availability are missing for 2318 observations across 10 hospitals.

**Figure 2.**
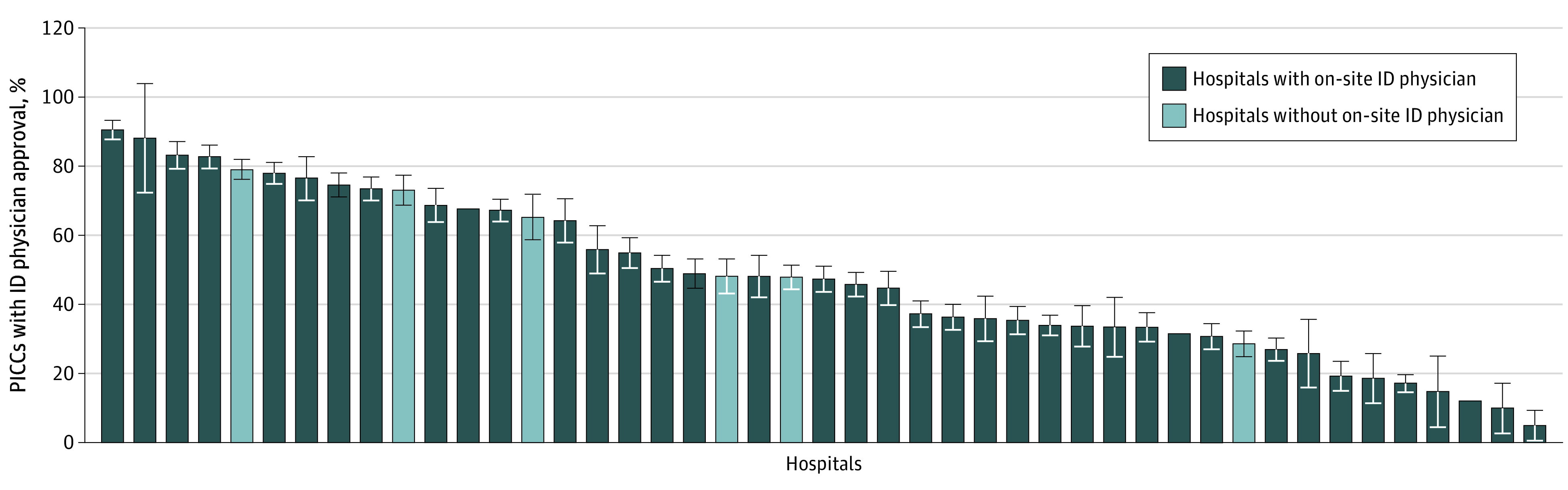
Proportion of Peripherally Inserted Central Catheters (PICCs) Placed for Intravenous Antimicrobials That Had Approval From an Infectious Disease (ID) Physician Prior to Placement, by Hospital (N = 21 317) Each bar represents 1 hospital. Not shown are 2 hospitals with less than 25 observations. Two hospitals also had no ID physician approvals (both lacked onsite ID physician availability). Error bars indicate 95% CIs.

All 42 hospitals had an antimicrobial stewardship program. Nearly three-quarters (73.8% [31 of 42]) of hospitals reported having onsite ID physicians, and 21.4% (9 of 42) had ID physicians who either physically visited the hospital or were available for remote consulting. Approval rates by an ID physician of PICCs placed with an indication for intravenous antimicrobials varied across hospitals from 1.8% (7 of 393) to 90.7% (388 of 428), including among those without onsite ID physicians (Figure 2). Hospital characteristics associated with ID physician approval are shown in Table 1.

Among PICCs with an indication for intravenous antimicrobials, those with ID physician approval were more likely to be single-lumen (87.0% [8908 of 10 238] vs 59.7% [6820 of 11 415]; *P* < .001), in place for more than 5 days (95.4% [9765 of 10 238] vs 85.8% [9792 of 11 415]; *P* < .001), and placed in patients without CKD (87.1% [8914 of 10 238] vs 83.3% [9503 of 11 415]; *P* < .001) (Table 2). Compared with PICCs without documented ID physician approval, those approved by an ID physician were more likely to meet all 3 appropriateness criteria (72.7% [7446 of 10 238] appropriate with approval vs 45.4% [5180 of 11 415] appropriate without approval). After adjustments, documented ID physician approval remained associated with overall appropriateness (OR, 3.53; 95% CI, 3.29-3.79) and each individual metric of PICC appropriateness.

**Table 2.** Data on PICC Appropriateness and Complications by ID Physician Approval

Outcomes	Documented ID physician approval before PICC insertion, No. (%)		Unadjusted OR (95% CI)	*P* value	Adjusted OR (95% CI)^a^	*P* value
	Yes (n = 10 238)	No (n = 11 415)				
All 3 appropriateness criteria met^b^	7446 (72.7)	5180 (45.4)	3.18 (3.01-3.70)	<.001	3.53 (3.29-3.79)	<.001
Single lumen	8908 (87.0)	6820 (59.7)	4.40 (4.12-4.71)	<.001	5.20 (4.78-5.66)	<.001
Used in eGFR ≥45 mL/min/1.73 m^2^	8914 (87.1)	9503 (83.3)	1.36 (1.27-1.47)	<.001	1.24 (1.13-1.36)	<.001
In place for >5 d	9765 (95.4)	9792 (85.8)	3.41 (3.07-3.79)	<.001	3.50 (3.11-3.94)	<.001
Major complication	665 (6.5)	1292 (11.3)	0.55 (0.50-0.61)	<.001	0.58 (0.52-0.65)	<.001
Catheter occlusion	432 (4.2)	976 (8.6)	0.48 (0.43-0.54)	<.001	0.51 (0.44-0.58)	<.001
DVT	148 (1.4)	238 (2.1)	0.68 (0.55-0.83)	<.001	0.72 (0.57-0.91)	.005
CLABSI	107 (1.0)	129 (1.1)	0.91 (0.71-1.16)	.43	0.96 (0.72-1.27)	.76

Abbreviations: CLABSI, central line–associated bloodstream infection; DVT, deep vein thrombosis; eGFR, estimated glomerular filtration rate; ID, infectious disease; OR, odds ratio; PICC, peripherally inserted central catheter.

^a^ Adjusted results were calculated using a logistic mixed-effect model that adjusts for patient age, sex, body mass index, race/ethnicity, Charlson Comorbidity Index score, hospital bed number, profit status, teaching status, and year of PICC placement, with hospital-specific random effects.

^b^ Full compliance with PICC recommendations indicates PICC device was single-lumen, was not inserted if patient’s eGFR was less than 45 mL/min/1.73 m^2^, and was not in place for 5 days or less.

In unadjusted analyses, documented ID physician approval prior to insertion was associated with lower odds of PICC-related complications (6.5% [665 of 10 238] with approval vs 11.3% [1292 of 11 415] without approval; OR, 0.55; 95% CI, 0.50-0.61; *P* < .001) (Table 2). Approval by an ID physician was associated with fewer of 2 major complications, catheter occlusion (OR, 0.48; 95% CI, 0.43-0.54; *P* < .001) and DVT (OR, 0.68; 95% CI, 0.55-0.83; *P* < .001). Owing to low rates of CLABSI, no difference in the odds of CLABSI was observed between groups (OR, 0.91; 95% CI, 0.71-1.16). Results were similar after adjustment.

At the facility level, hospitals in the highest quartile of ID physician approval rates for PICC placement demonstrated a higher overall level of PICC appropriateness (63.4% [3617 of 5709] vs 56.5% [9009 of 15 944]; *P* < .001) and lower complications (7.6% [433 of 5709] vs 9.6% [1524 of 15 944]; *P* < .001) than hospitals in the lower 3 quartiles of ID physician approval.

The association of ID physician approval with appropriateness and outcomes was robust to multiple sensitivity analyses, including models reporting results as relative risks rather than as ORs (eTable 1 in the Supplement) and models that were restricted to a single PICC placement in instances where patients received multiple devices (eTable 2 in the Supplement).

## Discussion

In this study of 21 653 PICCs placed for an indication for intravenous antimicrobials in 42 Michigan hospitals, documented ID physician approval of PICC placement prior to insertion was associated with a higher level of catheter appropriateness and fewer adverse events. Peripherally inserted central catheters approved by ID physicians were more often single-lumen catheters, placed in patients with normal kidney function, and with longer dwell times, suggesting that decision-making related to these devices was purposeful and deliberate. Taken together, these findings have important clinical and policy implications for the use and safety of PICCs placed for intravenous antimicrobials.

Although our study was not designed to address how ID physician involvement prior to PICC use may lead to differences in appropriateness and outcomes, 2 theories are plausible. First, it is possible that ID physicians (who routinely see patients receiving outpatient parenteral antimicrobial therapy) are more cognizant of device appropriateness; patients with PICCs approved by ID physicians were more likely to have single-lumen devices and less likely to have CKD. The higher use of single-lumen devices likely played a role in the association of ID physician approval with patient outcomes because single-lumen devices have lower rates of occlusion, DVT, and CLABSI.^18,19^ Second, it is possible that consulting ID physicians after PICC placement promulgates short-term PICC use. For example, if an ID physician finds that intravenous antimicrobials are unnecessary, that the risk of complications is high, or that oral therapy is appropriate, the ID physician may recommend removal of PICCs, leading to dwell time of 5 days or fewer.^20^ In contrast, obtaining ID physician approval prior to PICC placement may prevent PICC placement altogether. Because we track only patients for whom PICCs are placed and not those for whom this decision may be different, a prospective intervention would be necessary to test this hypothesis.

We found wide variation in the prevalence of ID physician approval prior to PICC placement. Some of this difference may be associated with the varying availability of consultations. For example, one hospital reported no access to ID physicians, and thus no approval by an ID physician prior to PICC insertion was granted. However, among the 73.8% of hospitals with access to onsite ID physicians, approval prior to PICC use varied widely, indicating that interhospital variability may not be explained by access to an ID physician alone. Another possible explanation is that PICC placement protocols may inform variation. For example, some hospitals may require ID physician approval prior to PICC placement or as part of outpatient parenteral antimicrobial therapy planning, leading to more appropriate device use.^21^ Also, there is likely to be some variation between hospitals with respect to when ID physicians are consulted. Studies have shown wide variation in the use of ID physician consultations and in the time to consultation in patients with bacteremia or transplant-related infections.^15,22^ Similarly, physician consultation practices vary (eg, intensivists may be less likely to consult an ID physician than other specialties).^23^ Thus, hospitals where ID physicians are engaged early and often may provide more opportunities for ID physicians to inform and improve PICC use. We also did not assess how ID physicians become involved in PICC approval or who within the ID team becomes involved. For example, it is possible that some antibiotic stewardship programs specifically include device assessment during antibiotic assessment. Similarly, ID-trained pharmacists may also offer additional insight into device use when providing recommendations on appropriate antibiotic therapy. Finally, patient characteristics may be associated with ID physician consultation and approval. For example, clinicians may be less likely to consult an ID physician if patients have multiple reasons for needing a PICC, beyond administration of intravenous antimicrobials. Patients with multiple indications for PICCs may have more complicated diseases and, therefore, may be at risk both for higher inappropriate PICC use and for more PICC-related complications. Thus, this group potentially has the most to benefit from ID physician involvement in PICC decision-making.

### Limitations and Strengths

Our study has limitations. First, we cannot say how approval from an ID physician was associated with PICC use; however, anecdotal discussions with our sites suggest that ID physician engagement is directly associated with PICC selection (eg, number of lumens) and device dwell times. Future qualitative studies using ethnographic methods might be useful in this respect. Also, we cannot assess whether ID physician consultation actually prevented PICC placement. Second, we operationalized ID physician approval by looking for documentation in medical records. It is possible that some PICCs may have been placed after verbal approval that was not documented; however, because this would bias our findings to the null (no effect due to misclassification), we would then underestimate the association of ID physician approval with PICC appropriateness and outcomes. Third, our study is observational and thus suggests an association rather than causation and may not be generalizable beyond Michigan. Fourth, because patients were not randomized to PICC placement, there is a potential for selection bias.

However, our study also has strengths, including robust statistical methods, inclusion of a large group of diverse hospitals, and the use of a number of sensitivity analyses to verify our findings. Second, we conducted a formal assessment of ID physician approval to ascertain the level of engagement of these physicians, a practice that is difficult to assess without manual medical record review. Third, we performed a rigorous assessment of appropriateness using validated criteria, lending strength and importance to our findings. Fourth, all complications were reviewed using strict definitions, ensuring that adverse events were appropriately recognized in a standardized fashion across sites.

Our study has important implications and raises the question of whether hospitals should enact policies that require ID physician approval of PICCs prior to placement, especially if the PICCs are inserted for administration of intravenous antimicrobials. This approach would be similar to existing policies requiring a nephrology physician’s approval for vascular catheter use in patients with CKD. Furthermore, by reducing expensive PICC-related complications and improving patient outcomes, ID physician approval prior to PICC placement could uniquely demonstrate the value of ID consultants to health systems. Such an approach would be consistent with guidelines and studies that have shown that ID physician involvement in outpatient parenteral antimicrobial therapy improves the level of appropriateness of care and patient outcomes.^24,25^

## Conclusions

This cohort study suggests that ID physician approval of PICC placement for administration of intravenous antimicrobials is associated with a higher level of device appropriateness and fewer complications. Policies that encourage ID physician engagement before placing PICCs for administration of intravenous antimicrobials may improve patient safety.

## Notes

Supplement. **eTable 1.** Peripherally Inserted Central Catheter Appropriateness and Complications by ID Approval, Sensitivity Analysis Showing Risk Ratios **eTable 2.** Peripherally Inserted Central Catheter Appropriateness and Complications by ID Approval, Sensitivity Analysis Including Only First PICCs Placed per Patient, N = 20,528
