# Supplementary material for: Association of Infectious Disease Physician Approval of Peripherally Inserted Central Catheter With Appropriateness and Complications
Source: JAMA Netw Open. 2020 Oct 21;3(10):e2017659. doi: 10.1001/jamanetworkopen.2020.17659 (PMC7578772; doi:10.1001/jamanetworkopen.2020.17659)
Supplement: Supplement. — eTable 1. Peripherally Inserted Central Catheter Appropriateness and Complications by ID Approval, Sensitivity Analysis Showing Risk Ratios eTable 2. Peripherally Inserted Central Catheter Appropriateness and Complications by ID Approval, Sensitivity Analysis Including Only First PICCs Placed per Patient, N = 20,528 [file jamanetwopen-e2017659-s001.pdf]

Supplementary Online Content

Vaughn VM, O'Malley M, Flanders SA, et al. Association of infectious disease physician approval of peripherally inserted central catheter with appropriateness and complications. *JAMA Netw Open.* 2020;3(10):e2017659. doi:10.1001/jamanetworkopen.2020.17659

eTable 1. Peripherally Inserted Central Catheter Appropriateness and Complications by ID Approval, Sensitivity Analysis Showing Risk Ratios

eTable 2. Peripherally Inserted Central Catheter Appropriateness and Complications by ID Approval, Sensitivity Analysis Including Only First PICCs Placed per Patient, N = 20,528

This supplementary material has been provided by the authors to give readers additional information about their work.

© 2020 Vaughn VM et al. *JAMA Network Open.*

eTable 1. Peripherally Inserted Central Catheter Appropriateness and Complications by ID Approval, Sensitivity Analysis Showing Risk Ratios

Outcomes	Documented ID approval before PICC insertion, N (%) (N = 10,238)	No documented ID approval before PICC insertion, N (%) (N=11,415)	Unadjusted odds ratio (95% confidence interval)	*P*	Adjusted odds ratio * (95% confidence interval)	*P*
All three appropriateness criteria met**	7,446 (72.7%)	5,180 (45.4%)	3.18 (3.01, 3.7)	<.001	3.53 (3.29, 3.79)	<.001
Single lumen	8,908 (87.0%)	6,820 (59.8%)	4.40 (4.12, 4.71)	<.001	5.20 (4.78, 5.66)	<.001
Used in eGFR ≥ 45	8,914 (87.1%)	9,503 (83.3%)	1.36 (1.27, 1.47)	<.001	1.24 (1.13, 1.36)	<.001
In place for > 5 days	9,765 (95.4%)	9,792 (85.8%)	3.41 (3.07, 3.79)	<.001	3.50 (3.11, 3.94)	<.001
			**Unadjusted relative risk ratio (95% confidence interval)**		**Adjusted relative risk ratio* (95% confidence interval)**	
Major complication***	665 (6.5%)	1,292 (11.3%)	0.58 (0.53, 0.64)		0.61 (0.55, 0.67)	
Catheter Occlusion	432 (4.2%)	976 (8.6%)	0.50 (0.45, 0.56)		0.52 (0.47, 0.59)	
DVT	148 (1.5%)	238 (2.1%)	0.68 (0.56, 0.83)		0.73 (0.60, 0.91)	
CLABSI	107 (1.1%)	129 (1.1%)	0.90 (0.71, 1.2)		0.99 (0.77, 1.3)	

* Adjusted odds ratios were calculated using a logistic mixed-effect model that adjusts for patient age, sex, BMI, race, Charlson score, hospital bed number, profit status, teaching status and year of PICC placement, with hospital-specific random effects.
** Adjusted relative risks were adjusted for patient age, sex, BMI, race, Charlson score, hospital bed number, profit status, teaching status and year of PICC placement using a log-binomial regression model
*** Full compliance with PICC recommendations indicates PICC device was single-lumen, was not inserted if patient's eGFR <45 mL/min/1.73 m^2 and was not in place for ≤5 days.
**** PICC-related major complications include CLABSI, DVT, or catheter occlusion.
Abbreviations: PICC, peripherally inserted central catheter; ID, infectious disease; eGFR, estimated glomerular filtration rate; DVT, deep vein thrombosis; CLABSI, central line associated blood stream infection; BMI, body mass index.

© 2020 Vaughn VM et al. *JAMA Network Open.*

eTable 2. Peripherally Inserted Central Catheter Appropriateness and Complications by ID Approval, Sensitivity Analysis Including Only First PICCs Placed per Patient, N = 20,528

**Outcomes**	**Documented ID approval before PICC insertion, N (%) (N =9,672)**	**No documented ID approval before PICC insertion, N (%) (N=10,856)**	**Unadjusted odds ratio (95% confidence interval)**	***P***	**Adjusted odds ratio* (95% confidence interval)**	***P***
All three appropriateness criteria met**	1231 (12.7%)	4405 (40.6%)	3.26 (3.07, 3.45)	<.001	3.60 (3.34, 3.87)	<.001
Single lumen	8441 (87.3%)	6451 (59.4%)	4.68 (4.36, 5.03)	<.001	5.46 (5.01, 5.96)	<.001
Used in eGFR ≥ 45	1250 (12.9%)	1815 (16.7%)	1.35 (1.25, 1.46)	<.001	1.23 (1.12, 1.35)	<.001
In place for > 5 days	446 (4.6%)	1549 (14.3%)	3.44 (3.09, 3.84)	<.001	3.55 (3.15, 4.01)	<.001
			**Unadjusted odds ratio (95% confidence interval)**		**Adjusted odds ratio (95% confidence interval)**	
Major complication***	615 (6.36%)	1224 (11.27%)	0.53 (0.48-0.59)		0.57 (0.51-0.64)	
Catheter Occlusion	404 (4.18%)	925 (8.52%)	0.47 (0.42-0.53)		0.5 (0.44-0.58)	
DVT	135 (1.4%)	225 (2.07%)	0.67 (0.54-0.83)		0.72 (0.57-0.91)	
CLABSI	98 (1.01%)	119 (1.1%)	0.92 (0.71-1.21)		0.95 (0.71-1.28)	

* Adjusted odds ratios were calculated using a logistic mixed-effect model that adjusts for patient age, sex, body mass index, race, Charlson score, hospital bed number, profit status, teaching status and year of PICC placement, with hospital-specific random effects.

** Full compliance with PICC recommendations indicates PICC device was single-lumen, was not inserted if patient's eGFR <45 mL/min/1.73 m^2 and was not in place for ≤5 days.

*** PICC-related major complications include CLABSI, DVT, or catheter occlusion.

Abbreviations: PICC, peripherally inserted central catheter; ID, infectious disease; eGFR, estimated glomerular filtration rate; DVT, deep vein thrombosis; CLABSI, central line associated blood stream infection.

© 2020 Vaughn VM et al. *JAMA Network Open.*
